# A biomedical decision support system for meta-analysis of bilateral upper-limb training in stroke patients with hemiplegia

**DOI:** 10.1515/biol-2022-0607

**Published:** 2023-07-27

**Authors:** Linna Jin, Zhe Yang, Zhaojun Zou, Tao Wu, Hongying Pan

**Affiliations:** Department of Nursing, Sir Run Run Shaw Hospital, Zhejiang University School of Medicine, No. 3, Qingchun East Road, Hangzhou, Zhejiang, 310020, China; Department of Rehabilitation Medicine, Sir Run Run Shaw Hospital, Zhejiang University School of Medicine, No. 3, Qingchun East Road, Hangzhou, Zhejiang, 310020, China; Department of Sleep Medicine, Sir Run Run Shaw Hospital, Zhejiang University School of Medicine, No. 3, Qingchun East Road, Hangzhou, Zhejiang, 310020, China

**Keywords:** stroke, bilateral upper-limb training, unilateral training, meta-analysis

## Abstract

The purpose of this study is to investigate the efficacy of bilateral upper-limb training (BULT) in helping people with upper-limb impairments due to stroke or brain illness regain their previous level of function. Patients recuperating from a stroke or cerebral disease were given the option of undergoing BULT or conventional training to enhance their upper-limb function. Participants were randomly allocated to one of the several different fitness programs. Results from the action research arm test, Box and block test, Wolf motor function test, Fugal–Meyer evaluation, and any other tests administered were taken into account. Some researchers have found that exercising with BULT for just 30 min per day for 6 weeks yields significant results. There were a total of 1,411 individuals from 10 randomized controlled trials included in this meta-analysis. Meta-analysis findings revealed that biofeedback treatment outperformed conventional rehabilitation therapy in reducing lower leg muscular strain, complete spasm scale score, electromyography score, and inactive ankle joint range of motion. An analysis of the literature found that BULT improved limb use in people who had suffered a stroke and hemiplegia but it did not provide any additional benefit over unilateral training.

## 1 Introduction

Stroke’s great prevalence and potential for long-lasting impairment make it a major public health concern worldwide [1]. About 55–75% of patients suffer from different degrees of upper-limb dysfunction at the early onset stage [2,3]. After 6 months of stroke, more than one-third of patients will have persistent upper-limb dysfunction [4,5], which significantly reduces the self-care ability of patients and brings physical and psychological distress to patients and their families [2]. In the United States, the medical expenses of stroke, including hospitalization, rehabilitation, and subsequent care, were estimated to be $140,048 per capita [6]. The burden of care is upcoming at an unprecedented rate, and the development of effective strategies must be considered a national priority [4]. The upper limbs are mainly characterized by flexible, coordinated, and skilled motor functions, all of which are involved in fine motor and complex activities in daily life. Stroke rehabilitation has a significant hurdle in the form of the patient’s slow and laborious road to recovery. Increasing the probability of functional recovery in the upper limbs is one of the most serious topics in clinical research [7]. Research has demonstrated that a patient’s handgrip strength on the “healthy side,” which some studies have indicated is not the unaffected side in stroke patients, is an independent predictor of functional recovery and prognosis during short-term post-stroke therapy [8,9]. After a stroke, the activities of patients’ healthy limbs are also significantly reduced, and the healthy limbs are always ignored.

In recent years, bilateral training (BT) has been widely used in rehabilitation stroke patients, which has a certain curative effect on improving the function of the hemiplegic upper limbs [10]. Bilateral upper-limb training (BULT) is a training method in which both upper limbs work together symmetrically or asymmetrically in different motor patterns. Symmetrical training mainly emphasizes the synchronous extension, flexion, grasping, rotation, and movement of healthy and affected upper limbs [11,12]. In asymmetric training, activities of daily living (ADLs) are usually coordinated by both bilateral upper-limb coordination, such as knotting, ring tossing, unscrewing bottle caps, folding towels, wiping tables, drinking water, tying shoelaces, zipping up and opening backpacks, and so on [13,14]. BULT can be used alone or in conjunction with other forms of training to improve motor function. These include Retro-Tongue-Base-Pharynx (RTBP), basic metabolic panel, bilateral arm training with rhythmic auditory cueing (BATRAC), virtual reality (VR), neuromuscular electrical stimulation (NMES), medical transcription (MT), etc. [15]. In addition, it can be used in different stages of stroke but there are some disputes regarding its training effect and intervention period. Moreover, the training time and period are quite different in the studies, and no unified standard has been established.

The comprehensive review includes BULT research that was proposed by Chen et al. [10] which included functional training, RTBP, and BATRAC. There was high heterogeneity among these studies, without further analysis or discussion on the different onset stages, training periods, or training doses. Lee et al. [13] introduces recovery of upper limb function after stroke: a comparison of the efficacy of BT and constraint-induced movement therapy (CIMT). High-intensity, daily exercise is performed on the injured upper limbs for at least 6 h, with reduced activity for the healthy limbs. BT may be a better alternative for improving ADLs than CIMT because of the fatigue and other problems that may occur with CIMT.

Researchers conducted a comprehensive literature evaluation to establish which of BT and UT is more effective for restoring muscular function in the upper extremities after stroke. Furthermore, we confirmed BULT’s effectiveness and revealed the best intervention and training periods and doses for optimal clinical practice and decision-making.

The Jintronix system employs Microsoft Kinect to help stroke survivors regain movement in their arms and hands by way of engaging and difficult exercises tailored to their specific skill level. As a result of using this exergame approach, therapists can track their patients’ quantitative improvement during therapy and make appropriate adjustments to their training regimen as needed. Including a point system in its exergames, this method has been shown to boost physical education outcomes. This is accomplished by giving the patient progressively more challenging tasks and giving them instantaneous input on how they are doing. The exergame system’s built-in tracking capabilities suggest that patients may soon be able to work out without the constant presence of a medical professional. Because of this, it may become possible to provide more telerehabilitation services to people in their homes and to expand access to rehabilitation services provided in clinics. To rephrase, there would be no need for extra staff to enable patients recovering from strokes to receive more comprehensive therapy programs. The feasibility, safety, and efficacy of this exergame approach as an adjunct to conventional therapy have yet to be determined; however, so further study is warranted.

The goal of this research is to determine how well BULT can assist stroke and other neurological disorder survivors recover their upper-body movement. Debate and analysis of the past were undertaken to discover the origins of the present. The outcomes of any tests administered, such as the action research arm test (ARAT), Box and block test (BBT), Wolf motor function test (WMFT), Fugl–Meyer evaluation, and others, were considered. About 24,000 pieces of literature were analyzed in this research. From the data, it is clear that there is a sizable gap in functional movement assessment (FMA) protections [16]. Mind-wrecking disruption hemiplegics who undergo BULT treatment report a higher level of satisfaction with their circumstances. Proponents of BULT exercise argue that even 30 min a day, 5 days a week for 6 weeks can have positive effects. The meta-analysis incorporated data from 10 randomized controlled trials (RCTs), with a total of 1,411 participants. The results of this meta-analysis show that biofeedback is more effective than traditional physical therapy at decreasing lower-limb muscle tension, total spasm scale score, electromyography score, and passive ankle joint range of motion.

## 2 Literature review

According to Rodgers et al. (2020) [17], stroke has an unusually heavy illness burden as it is one of the major worldwide causes of chronic disability and low quality of life. The inability to use one’s hands and arms effectively is a common complaint among stroke survivors. So, the major focus of stroke rehabilitation is to restore the ability to utilize the affected limbs, especially the arms and hands. Modern therapies for the hands and arms have come a long way, with some even using electromechanical components to aid with training. Due to its potential to deliver a challenging, regular, and repetitive therapy, electromechanically assisted arm training has been proposed as a feasible addition to evidence-based rehabilitation. Systematic evaluations suggest that arm exercise with electromechanical aid may boost motor function in the upper limbs, although this seems to have little therapeutic value. Although researchers could not prove it, they hypothesized that robotic-assisted arm training would negatively affect upper-body muscular tone.

In a previous study [18], it was argued that a prosthetic, such as an exoskeleton or robotic arm, may be used to move the joints of a paralyzed arm. Torque actuators, which provide rotating forces at a joint, might be included in the design of exoskeletons to facilitate mobility. The robotic arm may hold the patient’s arm in the horizontal plane as the patient moves their trunk, aiding in the development of shoulder and elbow coordination.

Wu et al. (2021) [19] described that an end effector is the last point of control on a robotic prosthetic, possibly near the end of the prosthetic limb. After utilizing these devices, patients may have improved mobility in their proximal arms as well. A little touch on the caregiver’s part is enough to accomplish this. In robotics and medicine, end effectors serve as mechanical appendages that perform the functions of human hands [20]. They are multipurpose since they allow for separate arm movement. A patient’s hands may rest on either of the two handles while they operate the end effector. The patients may practice pronating or supinating their forearms and wrists by simply switching the position of the handles. Modifying the sound is as easy as resetting the controls to their original positions. In “active” mode, the healthy arm of the patient is utilized to assist the robot in moving the injured arm, whereas in “passive” mode, the robot performs all the work alone.

According to a meta-analysis study conducted by Saposnik and colleagues, VR is effective in rehabilitating upper-extremity muscle function after stroke. VR was found to significantly improve the limb function (seven studies) and daily living tasks (three studies) compared to control groups. This result was accomplished by cherry-picking research directly linked to how well UEs work. Twelve studies were pooled together for a recent Cochrane Review and meta-analysis, and the results showed that VR improved ADL completion and upper leg function [20].

Based on their findings, the authors conclude that VR therapy has a greater effect on patient outcomes in all three ICF domains than traditional treatment methods. However, there is a dearth of data, including the best time and amount to administer the medication. In addition, research examining the perspectives of either individuals or healthcare providers on the debut of such instruments is scarce. Lange and her coworkers’ emphasis on finding enjoyable exercise routines was not enough to change this. Cylinder and Peoples interviewed and observed stroke therapy inpatients to assess the system’s efficacy. A large number of people who have used the Wii to assist in their recovery have reported increased stimulus, the availability of more difficult tasks, and general pleasure compared to their prior methods of therapy. Research has shown that VR and video games can aid in the recovery of stroke patients, and most of these studies agree that these findings are promising but preliminary. Studies of technology adoption have also demonstrated the significance of the user’s viewpoint in achieving desirable results [21].

## 3 Methods

### 3.1 Search strategy

Two separate reviewers searched the databases PubMed, the Cochrane Library, Embase, the Web of Science, CINAHL, Scopus, PEDro, SinoMed, CNKI, VIP, and Wanfang Data from their respective launch dates on October 31, 1996, to the present day for articles on bilateral upper-limb training of stroke patients (October 31, 2021). The database was first released to the public on October 31, 1996. Stroke, cerebrovascular accident, cerebrovascular sickness, CVA, cerebral strokes, brain vascular accident, hemiparesis, hemiplegia, bilateral exercise, bimanual exercise, and bilateral motor were some of the phrases we used to find relevant articles. The website’s Appendix 1 offers advice on how to study effectively.

## 4 Eligibility criteria

### 4.1 Types of studies

RCTs with BULT for hemiplegic stroke patients were included, and reports were found in both English and Chinese.

### 4.2 Types of participants

The following people were included in the study: (1) a confirmed diagnosis of a stroke using CT or MRI in the presence of symptoms; (2) older than 18 years; and (3) at different stages, including acute, subacute, and chronic phases. We also applied the following exclusion criteria: (1) not the first stroke; (2), lesions on both sides of the brain; (3) combined with other severe diseases; (4) unable to complete training due to cognitive or vision impairment; and (5) primary limb movement disorders.

### 4.3 Types of interventions

BULT (functional task training) was performed on stroke patients with hemiplegia to improve upper-limb function. The control group received routine training or unilateral upper-limb training (UULT). We also developed the following exclusion criteria for the intervention: (1) BT in both the experimental and control groups; (2) BT containing RTBP, NMES, MT, VR, and BATRAC; and (3) CIMT as the UT.

### 4.4 Types of outcomes

According to the disability classification of the World Health Organization [22], upper-limb assessment indicators can be divided into different levels, such as motor impairment (injuries related to the body structure), limited movement (ability related to the body function), and limited participation (ability related to ADLs). The study includes one or more of the following outcome indicators: After completing the FMA, we evaluated upper-limb functionality using the WMFT, BBT, and ARAT.

### 4.5 Patient and public involvement

No patient was involved.

### 4.6 Data collection and analysis

#### 4.6.1 Study selection

Two separate researchers used the aforementioned search strategy to identify relevant main documents. Our reviewers ran a quick Note Express title/abstract comparison to weed out duplicates. The full texts of the included articles were then assessed for eligibility for the review. When disagreements or issues developed between the two researchers, they discussed the situation and looked for ways to work together. A third, unbiased writer served as an arbitrator if a dispute could not be settled between the two original authors.

#### 4.6.2 Data extraction

Following a thorough review of the whole text, the research design was classified, and information was acquired from the published literature using the predetermined framework, including trial design, characteristics of the included studies, study population, treatments, end measures, and conclusions. The data extraction was done independently by two researchers. Conflicting opinions on how to extract data were addressed and settled. When necessary, a third author was brought in to aid.

### 4.7 Risk of bias assessment

Higgins et al. used the Cochrane risk of bias calculator to assess the level of methodological rigor of the RCTs [23]. The standard bias risk chart takes into account a variety of issues, including the generation of sequences, the suppression of distribution sequences, the use of blocking procedures, a lack of appropriate outcome data, and selective outcome reporting. The chart included, in the first column of each column, a short description of each event that was discussed in this research, followed by the author’s evaluation of how successfully that event helped accomplish the objective of eradicating discrimination.

### 4.8 Data synthesis and analysis

We utilized RevMan5.3 to perform the statistical analysis (Cochrane Collaboration, version 5.3.4). The range of potential events was analyzed and quantified using both the Chi-square and the *I* ^2^ tests. When the *I* ^2^ number was below 50%, a fixed-effect model meta-analysis was performed. All studies with an *I* ^2^ number higher than 50% were analyzed using effect models in the meta-analysis. To analyze the data, we used a weighted mean difference (WMD) method and evaluated the signs for consistency on a scale. When comparing continuous data on varying dimensions, the standard mean difference (SMD) is a useful statistical measure. Statistical importance was assumed if the *p*-value was less than 0.05.

### 4.9 The rehabilitation exergaming system

Exergaming, a type of counseling, was used with the Jintronix system as part of this study. The company collaborated with a wide range of healthcare workers, including clinicians, doctors, and patients, to ensure that this system is intuitive and widely available. Using the Kinect sensor and without the need for any marks, this setup is an interactive video game for physical activity. Unlike the Nintendo Wii, this system does not necessitate a separate handheld gadget to track a player’s motion. A Kinect sensor is integrated into the exergaming system for recovery purposes so that players’ upper- and lower-body motions can be tracked in real time. This is achieved whether the musician is sitting, standing, holding, or even wearing their instrument. After capturing the player’s motions, the data are presented in the setting of a healing video game. The method allows for unidirectional and bilateral UE exercises to be performed on any surface and at various levels. The therapist can adjust the game’s pace, goal size, accuracy, and uniformity to meet the needs of the patient.

### 4.10 Study procedure

All of the study subjects had suffered a stroke and were enrolled in outpatient therapy at one of two facilities. During the therapy, they were able to take advantage of vocational therapy and/or physical recovery programs. Based on the patient’s individual needs, the interdisciplinary team saw them twice or three times per week, with the bulk of the time spent on vocational and physical therapy. In addition to traditional physical treatment, those in the control group also played exergames designed to improve leg muscle. Stroke survivors in the control group used the exergame system for 4 weeks, twice or three times a week, for 30 min each practice (not including setup and other contacts with the system). We ensured that they had opportunities for relaxation in between their gameplay and counseling appointments. Each location also had a clinician familiar with the therapy exergaming method present during the exergaming session. This medical professional was responsible for all of the clinic’s clients [24].

### 4.11 BULT is helpful in the case of stroke patients

Training with both arms and “bilateral arm training” has been shown to increase the activity in both the impacted and uninjured parts of the brain. This, in turn, may promote the recovery of stroke-harmed brain cells.

### 4.12 BULT therapy for stroke victim’s work in practice

First, the trainer focused on the basics, like getting out of bed, having a shower, and eating a healthy breakfast. When patients took part in routine tasks, they regained movement and their ability to operate in everyday living. Recovering from a stroke is made easier with the help of flexible technology, as shown by the research.

## 5 Results

### 5.1 Search process

**Figure 1:**
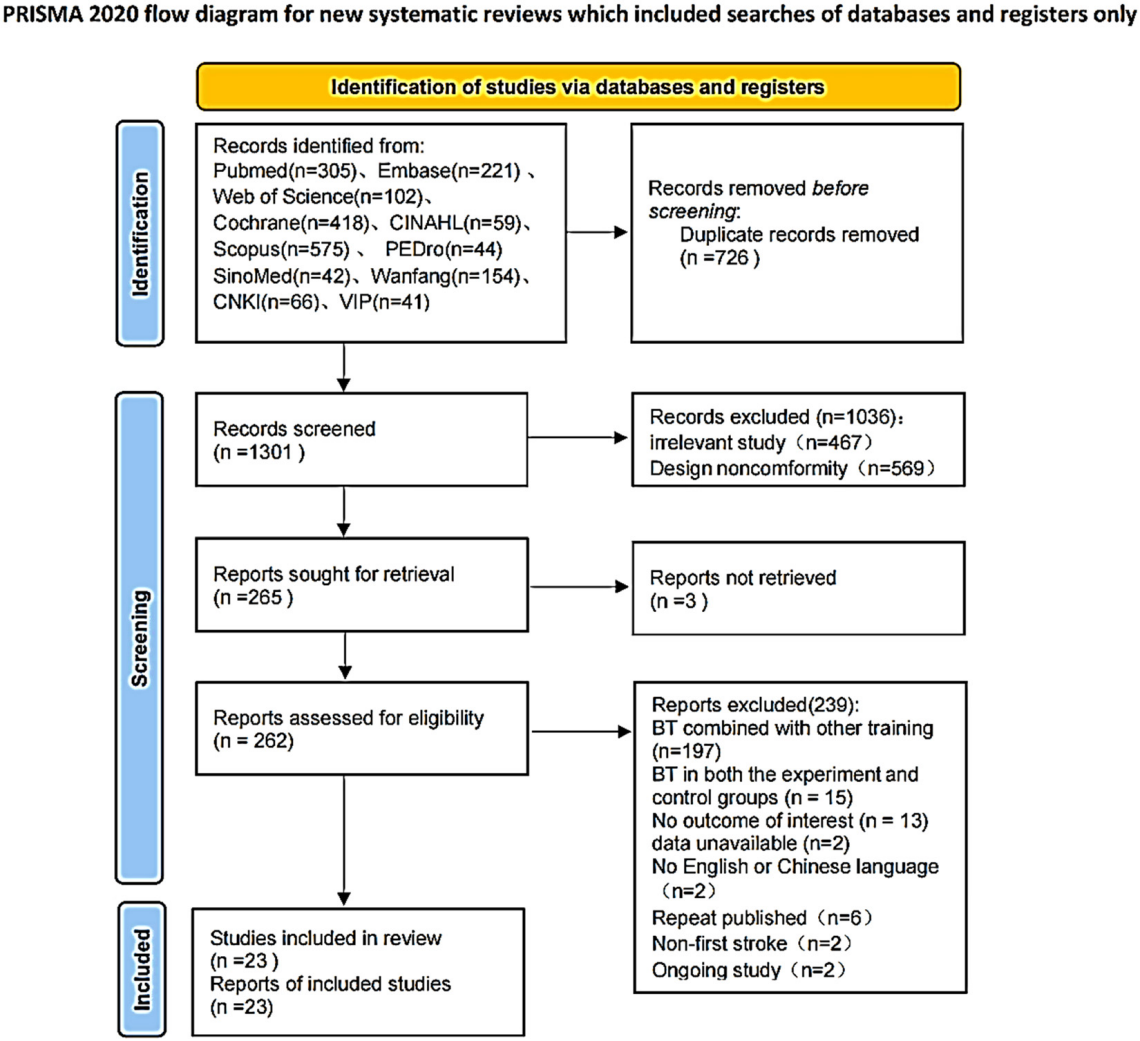
PRISMA search flow diagram.

According to the article retrieval strategy, we obtained 2,027 articles. After reference tracking, repeated screening, and independent screening by two researchers, 23 RCTs were finally included in the meta-analysis. Figure 1 shows the flow chart for article screening.

### 5.2 Study characteristics

Table 1 presents an overview of the primary features shared by the studies that were taken into consideration. In this section, we provide a detailed introduction to each study’s first author, year, research fields, sample size, intervention, and outcome mea sure, as well as general information including participant age, gender, primary illness, lesion location, and start time.

**Table 1:** Types, durations, frequencies, and results were all quantified in the studies that were included

Study (Country/year)	Participate (M/F)	Age ± SD	Type (CI/ICH)	Duration (month ± SD)	Intervention type	Duration of therapy	Study outcome
Desrosiers et al. (Canada/2005) [25]	BT: 9/11	BT: 72.2 ± 10.8	BT: 19/1	BT: 34.2 ± 34.4	BT: Examples of such activities include rolling out dough, opening and closing locks, measuring dry ingredients using a teaspoon, and folding towels	45 min/d, 5 d/wk, 4 wk	①②
	UT: 10/11	UT: 74.3 ± 10.1	UT: 21/0	UT: 35.4 ± 33.7 (days)	UT: Conventional therapy for the affected upper extremity		
Dhakate and Bhattad (India/2020) [12]	BT: 20	54 ± 10	Not reported	1–6 (mo)	BT: Lifting a shoebox overhead and lowering it again, pulling a chair, and lifting two polystyrene cups simultaneously and then putting them back	45 min/d, 5 d/wk, 4 wk	①
	UT: 20						
					UT: Conventional therapy for the affected upper extremity		
Easow et al. (India/2019) [26]	BT: 10/5	BT: 60.00 ± 9.35	BT: 15/0	Acute	BT: Clasping the hands, then moving to mouth, the elevation of the arms, turning toward sound side keeping shoulder well forward	20 min/d, 6 d/wk, 4 wk	③
	UT: 10/5	UT: 60.86 ± 9.96	UT:15/0		UT: Standard care treatment for affected upper limbs		
Han et al. (Korea/2016) [27]	BT: 5/8	BT:78.8	Not reported	BT: 83.0	BT: Ring hanging, desk cleaning, and drinking are all examples of bilateral actions	30 min/d, 5 d/wk, 6 wk	②
	UT: 3/9	UT:72.9(avg)		UT: 77.8 (avg)	UT: Same as the bilateral group but performed with impaired arm		
He et al. (China/2016) [28]	BT: 11/7	BT: 52.82 ± 11.25	BT: 11/7	BT: 24.52 ± 10.2	BT: Flexion, extension, adduction, and rotation of elbow and wrist joints, and hand grasping training	40 min/d, 5 d/wk, 12 wk	①
	UT: 10/8	UT: 50.37 ± 11.0	UT: 12/6	UT: 22.47 ± 9.17	UT: Just like the bilateral group, this one exclusively included the affected hand		
Kaur et al. (India/2019) [29]	Not reported	BT: 52 UT: 50 (avg)	13/3	2–16 (wk)	BT: Holding a lock and unlocking it, holding a bowl and picking stones from it, pulling a rope, tying a shoelace while keeping the elbows in the extended position, and opening a drawer	30 min/d, 5 d/wk, 4 wk	①③
					UT: Motor relearning program with impaired arm		
Kumar et al. (India/2011) [43]	BT: 9/6	BT: 66.67 ± 12.27	Not reported	Not reported	BT: Passive/active movements of all the joints of upper limbs, weight-bearing, supportive reaction, reaching activities than grasping, holding, and releasing activities; lastly performed ADL activities	45 min/d, 5 d/wk, 4 wk	①
	UT: 9/6	UT: 67.55 ± 9.69			UT: Same as the bilateral group with the affected UE		
Lee et al. (Korea/2017) [30]	BT: 9/6	BT: 57.33 ± 9.88	BT: 8/7	7–12 mo: BT (4); UT (5); 13–24 mo: BT (3); UT (5); ≥25 mo: BT (8); UT (5)	BT: Some common household tasks include washing dishes, composing letters, chopping vegetables, and folding clothes	30 min/d, 5 d/wk, 8 wk	①②
	UT: 10/5	UT: 54.60 ± 16.03	UT: 9/6		UT: General occupational therapy		
Lee et al. (Korea/2019) [31]	BT: 6/9	BT: 61.53 ± 8.81	Not reported	BT: 3.60 ± 1.30	BT: Wiping table with a towel, mimicking drinking, and moving blocks to boxes	30 min/d, 5 d/wk, 4 wk	①
	UT: 7/8	UT: 62.00 ± 8.13		UT: 3.20 ± 1.37	UT: General upper-limb rehabilitation		
Zhenjing et al. (China/2019) [11]	BT: 7/9	BT: 45.38 ± 9.78	BT: 12/8	BT: 4.63 ± 1.59	BT: Stacking blocks, placing wooden nails, turning over chess pieces, folding towels, unscrewing bottle caps, simulating drinking water, and throwing and catching the ball	60 min/d, 5 d/wk, 4 wk	①
	UT: 7/8	UT: 49.67 ± 10.6	UT:11/10	UT: 3.40 ± 1.5	UT: Same as the bilateral group but performed with the affected hand		
Lin et al. (Taiwan, China /2010) [32]	BT: 10/6	BT: 52.08 ± 9.60	Not reported	BT: 13.94 ± 12.73	BT: One needs both hands and a cup to do just about anything: watering plants, playing checkers, picking up dry beans, folding towels, handling huge screws or coins, etc	120 min/d, 5 d/wk, 3 wk	①
	UT: 9/8	UT: 55.50 ± 13.17		UT:13.12 ± 8.13	UT: General upper limb rehabilitation with neurodevelopmental techniques		
Lin et al. (Taiwan, China/2015) [33]	BT: 12/4	BT: 52.63 ± 10.49	BT: 9/7	BT: 27.75 ± 19.04	BT: By gradually raising or decreasing their grip strength with both hands, participants in a bilateral isometric handgrip force training program mimicked the intended force action	30 min/d, 3 d/wk, 4 wk	①④
	UT: 16/1	UT: 57.47 ± 10.29	UT: 6/11	UT: 21.82 ± 21.66	UT: Routine clinical rehabilitation is generally helpful for people with upper-limb hemiplegia		
Manjula et al. (India/2020) [44]	BT: 15	40–70	Not reported	>6 (mo)	BT: Wiping the table, reaching and placing objects, moving an object from table to shelf, bilateral elbow extension during horizontal reach, grasping an empty glass, taking it to mouth, and returning to starting position	Each activity was repeated 30 times, 4 wk	③
	UT: 15				UT: Analogous to the unilateral class but with a broken hand		
Meng et al. (China/2017) [34]	BT: 34/30	BT: 55.38 ± 6.97	BT: 50/14	BT: 8.87 ± 2.69	BT: Haptic perception training, bimanual coordination training, and functional training of the hands	60 min, 2 times/d, 5 d/wk, 2 wk	①③
	UT: 31/33	UT: 55.19 ± 7.82	UT: 45/19	UT: 9.08 ± 2.35 (h)	UT: Conventional rehabilitation training		
Morris et al. (England/2008) [35]	BT: 34/22	BT: 67.9 ± 13.1	BT: 3/53	BT: 22.6 ± 5.6	BT: To move a doweling peg from the tabletop to attach to the underside of a shelf; to move a block from the table onto a shelf; to grasp an empty glass, take it to the mouth and return to starting position; to point to targets raised	20 min/d, 5 d/wk, 6 wk	③
	UT: 27/33	UT: 67.8 ± 9.9	UT: 6/44	UT: 23.2 ± 5.7 (d)	UT: The wounded hand is used in a manner analogous to the bilateral group		
Renner et al. (Germany/2020) [36]	BT: 16/19	BT: 63.70 ± 13.38	BT: 13/22	BT: 35.17 ± 11.03	BT: Cycling movements of the arm, including forearm and wrist extension/flexion in passive–passive, motor-assisted, and actively moving against the resistance mode	20 min, 2 times/d, 5 d/wk, 6 wk	①
	UT: 16/18	UT: 63.34 ± 12.49	UT: 11/23	UT: 37.22 ± 13.56	UT: Same as the bilateral group but performed with the affected hand		
Song et al. (China/2020) [37]	BT: 35/24	BT: 44.47 ± 8.41	BT: 33/26	BT: 99.54 ± 43.9	BT: Flexion, extension, adduction, and rotation of elbow and shoulder joints	2 times/d, 6 d/wk, 4 wk	①
	UT: 37/24	UT: 44.00 ± 7.90	UT: 37/24	UT: 92.26 ± 39.28 (d)	UT: Same as the bilateral group, only with the injured hand		
Stoykov et al. (America/2020) [38]	BT: 7/1	BT: 61 ± 7.60	Not reported	BT: 62.9 ± 50.00	BT: Continuous symmetrical wrist flexion and extension	60 min/d, 5 d/wk, 6 wk	①
				UT: 68.13 ± 51.11	UT: Played computerized Jeopardy games with the affected hand		
	UT: 6/2	UT: 63 ± 5.21					
Tanavarapu et al. (India/2019) [39]	BT: 33	BT: 54.3	Not reported	Not reported	BT: Picking up a coin and manipulating it, catching a ball, wiping the table with a towel, elbow extension during horizontal reach, lifting a glass to drink, picking up a spoon to feed, and reaching forward/upward for a cup and replacing it	30 min/d, 5 d/wk, 4 wk	①
	UT: 32	UT: 55.3			UT: Same as the bilateral group, only with the injured hand		
Wang et al. (China/2015) [40]	BT: 16/10	BT: 51.2 ± 10.1	BT: 14/12	BT: 2.8 ± 0.7	BT: Flexion, extension, adduction, and rotation of elbow and wrist joints, hand-grasping training	30 min, 2 times/d, 5 d/wk, 6 wk	①
	UT: 17/9	UT: 50.3 ± 11.5	UT: 17/9	UT: 3.0 ± 1.0	UT: Same as the bilateral group but performed with the affected hand		
Wang et al. (China/2016) [45]	BT: 18/9	BT: 51.3 ± 5.6	BT: 16/11	BT: 3.0 ± 0.8	BT: Flexion, extension, adduction, and rotation of elbow and shoulder joints	60 min/d, 5 d/wk, 4 wk	①
	UT: 17/10	UT: 50.9 ± 5.5	UT: 14/13	UT: 2.9 ± 0.6	UT: Same as the bilateral group but performed with the affected hand		
Wei et al. (China/2017) [41]	BT: 6/4	BT: 62.0 ± 6.87	BT: 10/0	BT: 18.70 ± 4.03	BT: All the joints of upper-limb flexion, extension, abduction, and rotation; hand grasping training	30 min, 1time/d, 30 d	①
	UT: 5/5	UT: 60.70 ± 5.37	UT: 10/0	UT: 20.00 ± 4.05	UT: Same as the bilateral group but performed with the affected hand		
Wu et al. (Taiwan, China/2011) [42]	BT: 18/4	BT: 52.22 ± 10.72	Not reported	BT: 15.92 ± 13.74	BT: Lifting two cups, picking up two pegs, grasping and releasing two towels, and wiping the table with two hands	120 min/d, 5d/wk, 3 wk	④
	UT:16/6	UT: 55.19 ± 2.50		UT: 17.77 ± 12.45	UT: Rehabilitation routines that target the affected limb’s range of motion, stability, and weight bearing		

① FMA. ② BBT. ③ ARAT. ④ WMFT. BT, bilateral training; UT, unilateral training; M, male; F, female; CI, cerebral infarct; ICH, intracerebral hemorrhage; h, hour; d, day; wk, week; mo, month; avg, average.

### 5.3 Critical appraisal of quality

**Figure 2:**
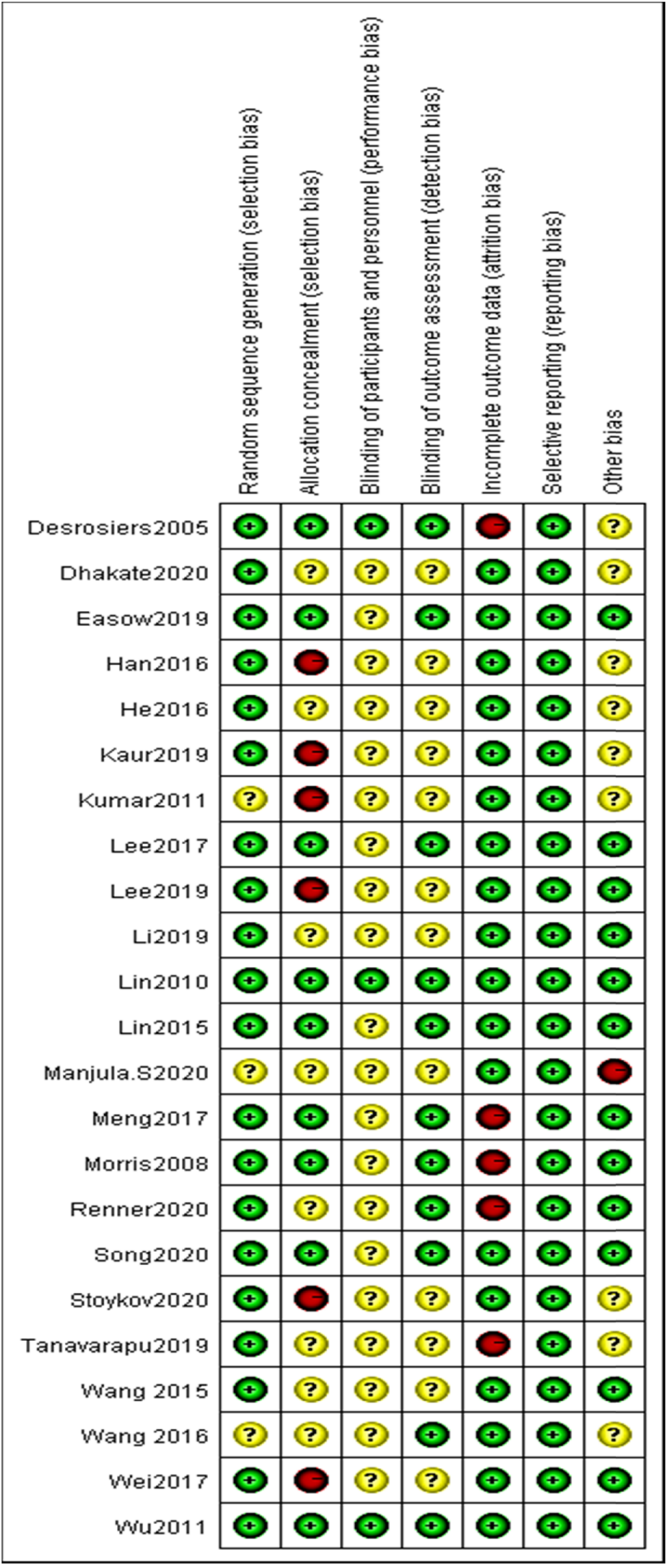
Risk-of-bias summary: authors’ judgments regarding the risk of bias of each item in each included study.

The findings of the assessment of the primary methodological quality indicators are presented in Figure 2 for the original articles that were taken into consideration. Twenty articles [11,12,25,26,27,28,29,30,31,32,33,34,35,36,37,38,39,40,41,42] had low risk in the random allocation methods, whereas three articles [43,44,45] did not describe the particular random allocation methods. In total, 20 articles [11,12,25,26,27,28,29,30,31,32,33,34,35,36,37,38,39,40,41,42] had low risk in the random allocation methods. In addition, it was discovered that 9 of the articles had limited potential for allocation suppression; 3 of the articles used blindfolded methods for the patients; 11 of the articles used blinded methods to evaluate the findings; and 5 of the articles documented the loss of samples. There was no selective reporting of the findings in any of the 23 articles, and 9 of the articles displayed other ambiguous sources of prejudice. These publications, on the whole, had a standard level that was somewhere in the middle.

### 5.4 Outcome analysis

#### 5.4.1 Motor impairment meta-analysis

The FMA has been utilized in 18 different research investigations to quantify hand and arm motor dysfunction. The high degree of variety was reflected in an *I* ^2^ score of 82%. The random-effects model showed that the BT group had a greater FMA than the UT group (MD = 5.80, 95% CI = [4.13–7.47], *p* = 0.00001). For this experiment, the *p*-value was so low that it could not even be measured in decimal places. In the FMA meta-analysis, no significant publication bias was found according to the Egger test (*p* = 0.929).

**Figure 3:**
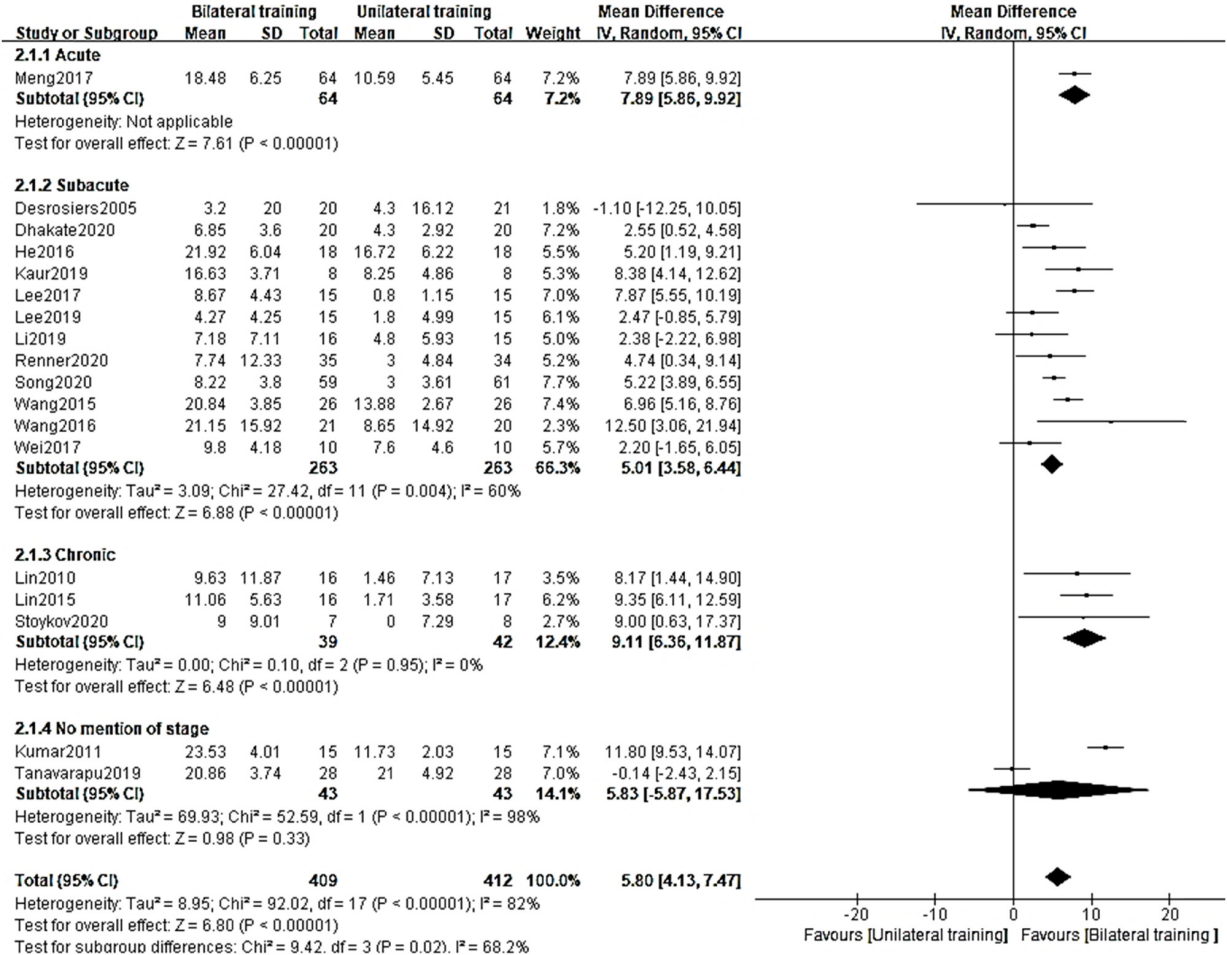
Subgroup analysis in the BT group and UT group at different stages.

The length of time after the stroke was a statistically significant differentiator between the BT and UT groups (MD = 7.89, 95% CI = [5.86–9.92], *p* = 0.00001; MD = 5.01, 95% CI = [3.58–6.44], *p* = 0.00001, *I* ^2^ = 60%; MD = 9.11, 95% CI = [6.03–11.01], *p* = 0.00001, *I* ^2^ = 60%) (6.36–11.87) (Figure 3).

**Figure 4:**
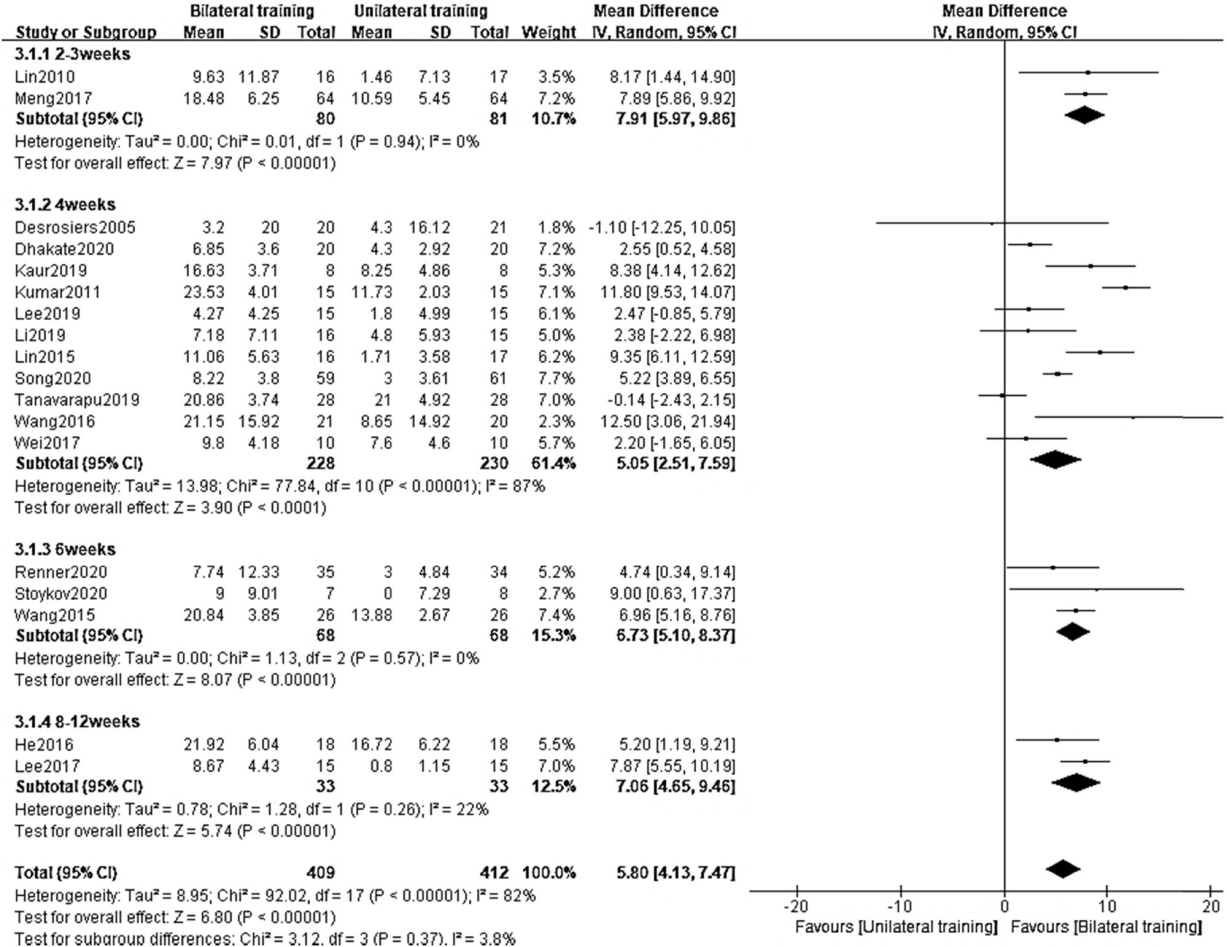
Subgroup analysis in the BT group and UT group at different training periods.

When compared with the UT group, the BT group had significant improvements in the subgroups based on training periods (2–3 weeks: MD = 7.91, 95% CI = [5.97–9.86], *p* < 0.00001, *I* ^2^ = 0%; 4 weeks: MD = 5.05, 95% CI = [2.51–7.59], *p* < 0.0001, *I* ^2^ = 87%; 6 weeks: MD = 6.73, 95% CI = [5.10–8.37], *p* < 0.00001, *I* ^2^ = 0%; 8–12 weeks: MD = 7.06, 95% CI = [4.65–9.46], *p* < 0.00001, *I* ^2^ = 22%, Figure 4).

**Figure 5:**
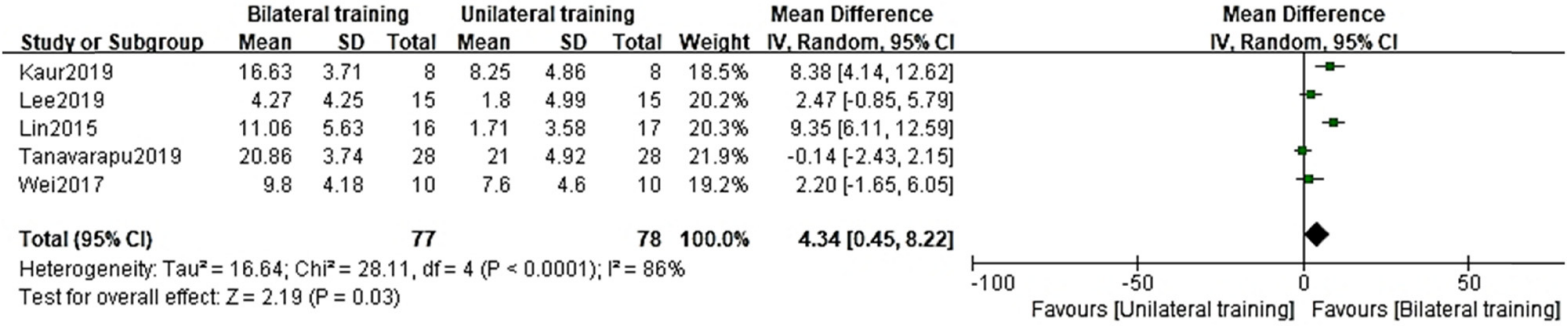
Forest plot of the FMA in the BT group and UT group with training 30 min per day.

There was a significant amount of variation (*I* ^2^ = 86%) between the findings of the five different studies on the benefits of getting at least half an hour of exercise daily. When a random-effects model was applied to the overall effect values, we were able to determine that the BT group had a significantly higher FMA than the UT group (Figure 5; MD = 4.34, 95% CI = (0.45–8.22), *p* = 0.03). Eliminating individual research has not benefited all that much in terms of bringing down the variation.

**Figure 6:**
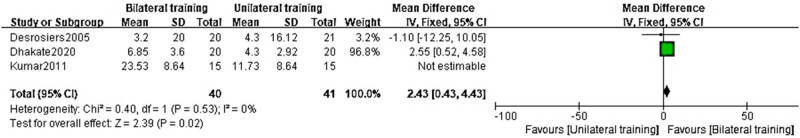
Forest plot of the FMA in the BT group and UT group with training 45 min per day.

Three publications reported exercise for 45 min each day had a high degree of inconsistency (*I* ^2^ = 95%). When the article by Kumar [43] was taken out of the mix, the level of heterogeneity dropped to 0%. This research described ADL-related coordination training in addition to inactive and active symmetrical training of both the left and right upper extremities. This could be the source of the heterogeneity in the results (Figure 6).

**Figure 7:**
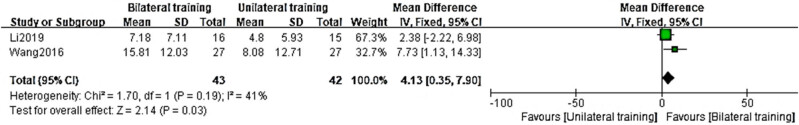
Forest plot of the FMA in the BT group and UT group with training 60 min per day.

The amount of variation in daily exercise activities lasting 60 min or longer was modest (*I* ^2^ = 41%) across the two studies. A fixed-effect model was utilized to conclude that there was a statistically significant effect size (MD = 4.13, 95% CI = [0.35–7.90], *p* = 0.03) (Figure 7).

#### 5.4.2 Functional performance meta-analysis

**Figure 8:**
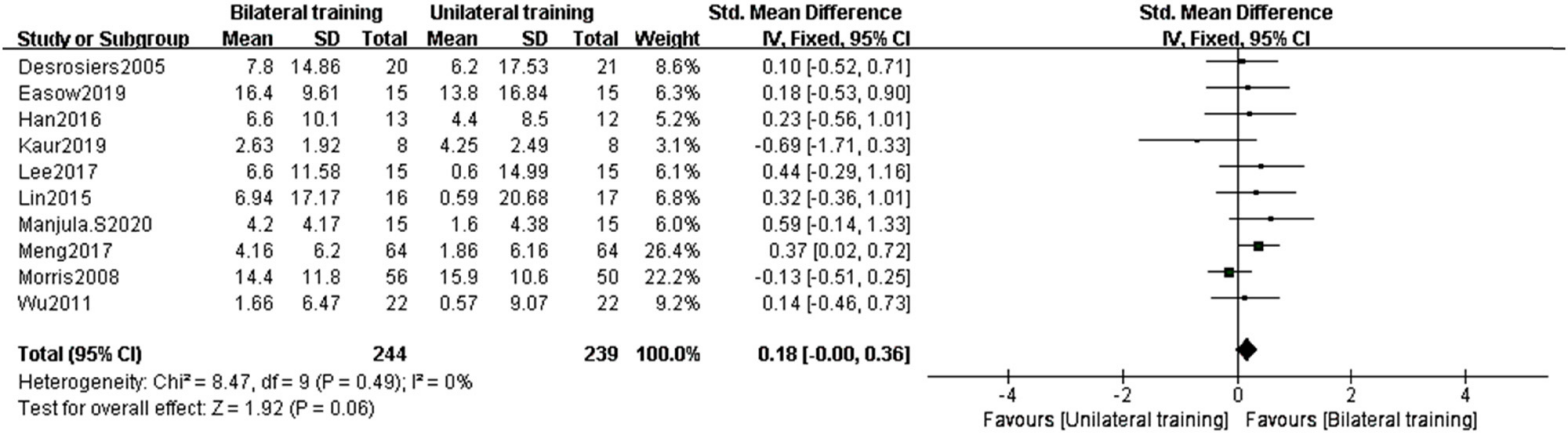
Forest plots of WMFT, ARAT, and BBT analysis in the BT group and UT group.

A comprehensive review and meta-analysis were able to substantiate that BULT is successful in recovering upper-limb function in patients who had suffered hemiplegic strokes. The FMA-UE of BT and UT was evaluated across 18 studies in this comprehensive review and meta-analysis for patients with movement disabilities in their upper extremities as a consequence of stroke. Our data and the data from other investigations demonstrate that the FMA-UE has made significant progress since it was first developed (Figure 8).

People who had suffered a stroke and were now feeling better agreed to take part in the research. As a result of the difficulty of the research, five of the initial subjects dropped out before it was completed. Four individuals in the intervention group and one person in the control group dropped out before the initial assessment, despite having given their full consent. The absence of time, energy, or inspiration was the typical cause. One participant in the intervention group was unable to participate in post-intervention and follow-up assessments due to mobility limitations brought on by capsulitis. The experts were taken aback by this finding. Although information about this patient’s participation in the study’s mortality phase was included in the end report, it was omitted from the efficacy report. Eighteen stroke victims had their concluding days monitored. Stroke victims and research participants with identifiable features were chosen.

Disputes surfaced after exploring different options. At least 8 lessons were spaced out over 4 weeks, with the average length of each session being 44 min. The typical amount of time spent playing an exergame is around 22 min. About half of the time was spent performing the actual work, and the other half of the time was spent on things like orientation, training, data collection, analysis, fine-tuning, and downtime. Only about half of the allotted time was used for the exercise. Participants’ success ranged from 32 to 64%. Participants in the control group reported no negative side effects. After a complete day of labor, the average VAS pain score was 2.2 out of 9.9, while the average Borg score was 3.6. No injuries were recorded during training, and only one person mentioned feeling confused. Both participants rated their level of pain and fatigue at 1.8 out of 9.

## 6 Discussion

Patients with hemiplegia after a stroke benefited from BULT, as shown by a meta-analysis and systematic review. Patients with motor impairment in their upper limbs as a result of stroke were the focus of 18 studies that analyzed the FMA-UE of BT and UT. Our findings corroborated those of the earlier research, showing substantial improvement in the FMA-UE [10]. Some studies demonstrated that bilateral movement can enhance the activation and connection of the main motor area M1, the primary sensory area S1, and the supplementary motor area of the bilateral cerebral hemispheres, which can dominate the movement of the homologous muscle groups [46,47]. Neuroplasticity may also involve the corticospinal system after a stroke. Most of the corticospinal axons pass through the contralateral side at the medullary cone level and dominate the contralateral limbs. However, about 10–20% of nerve fibers descend directly rather than cross to form the anterior corticospinal tract and dominate the ipsilateral limbs [9,48].

Rehabilitation after a stroke usually happens in four phases: the acute phase, the subacute phase, the late subacute phase, and the chronic phase [48]. Patients with acute, subacute, and chronic strokes all showed greater FMA-UE improvement in the BT group compared to the UT group, as determined by subgroup analysis. It was shown that the acute and subacute phases were more influential than the chronic period. Since the articles are still in the “subacute” or “subacute late” stages, there is a contradiction between them (7 days to 3 months and 3 to 6 months). One possible explanation for the paucity of research into the acute phase is the high risk of complications and overall poor health that characterize it [49]. In the early recovery from stroke, the activation of motor areas in both hemispheres increased, which was more obvious in the contralateral hemisphere, and then gradually decreased in the subsequent treatment [47,50]. A study reported that the spontaneous biological recovery of the brain is the most significant in the first 3 months after stroke onset and gradually weakens and stabilizes after 6 months [19]. Borschmann et al. [47] conducted a longitudinal study that evaluated the upper-limb function at 14 days, 6 weeks, and 3, 6, 12, 18, and 24 months after stroke and found that the most significant improvement occurred at 2–6 weeks after stroke. In addition, some patients showed improvement at 12–18 months, and several patients still presented with improvement at 24 months, which is consistent with Lang’s study [48]. Thus, BULT could be performed early if the patient is stable and can cooperate. Ambreen et al. [49] compared the effect of BULT on the upper-limb function in a patient who had left and right hemisphere damage, and the intergroup analysis showed no statistically significant difference. In addition, Renner et al. [36] reported that the FMA-UE and grip strength of the affected hands of patients with subcortical lesions after BT were much better than those after UT, but the situation of patients with cortex involvement was different. The lesion site and degree of stroke might also influence factors that should be considered in BULT. Other articles did not mention the specific lesion sites or grouping. Therefore, this study failed in the analysis and discussion of the relevant subgroup.

In addition, our subgroup analysis showed that the BT had a statistically significant effect on the FMA-UE of stroke patients with hemiplegia in different training periods compared with UT. Five articles used a 6-week training period without heterogeneity in these articles. Compared with the studies that used a training time of 2–3, 4, and 8–12 weeks, 6 weeks effect sizes increased, while the heterogeneity was lower. Therefore, the best effect was observed at 6 weeks. The results of Savant [50] also showed that compared with routine training after 4 weeks, isometric movement improved the functions of the shoulder abductor and external rotator, elbow flexor, and forearm pronator on the affected side, and these improvements were more obvious at 6 weeks, with statistically significant differences. Another review concluded that the median of limb rehabilitation training after a stroke was 6 weeks [51]. For training 120 min per day, 5 days per week, the training cycle lasted less than 3 weeks in two studies [32]. It is reported that with the extension of daily training time, patients were prone to fatigue and found it challenging to insist on long-term training [13]. However, the studies of long-term training were not enough in our research, and 6 weeks may not be the only influential factor. We need more extensive sample size trials to confirm its long-term effects.

Patients with mildly diverse strokes were given a BULT for 4 weeks in 11 studies. Furthermore, we investigated how everyday exercise time affected the BULT. The results of this research demonstrated that the FMA-UE could be enhanced through daily training sessions of 30, 45, or 60 min. The impact values were comparable across groups, suggesting that adding more exercise time per day would not yield any additional gains. Instead of documenting training hours, one research detailed the training procedures and schedule [37]. Nerve rearrangement and exercise outcomes were influenced by training intensity (rehabilitation dosage), but the optimum therapy dose and intensity limits are still unclear [52,53]. Stroke patients in the low-dose and high-dose groups who received upper-arm training with a robot in conjunction with vocational therapy showed modest but clinically significant improvements in FMA-UE ratings, according to Pila et al. [54]. High-dose and low-dose groups had similar rates of manual function improvement in the upper limbs [55]. A systematic review compared the effects of high-dose and low-dose single-rehabilitation training on limb injury and activity limitation of patients after stroke, which reported there were no statistically significant differences in the effects of weekly training for <5, 5–10, and >10 h per week on post-stroke upper-limb function, lower-limb function, or ADLs. This discovery suggested the existence of a therapy “optimal dose,” beyond which the advantages of further treatment diminished. As with the frequency and length of workouts, a patient’s endurance and exhaustion indicator will guide what kind of exercise regimen is best for them. Patients’ collaboration, interest, and desire must be taken into account during each recovery training [56,57]. Our research suggests that people who have recovered from a stroke should do BULT exercises for at least 30 min daily. This result should be taken with care, however, because sensitivity analysis cannot determine the actual reason for the difference.

When comparing the functional increase of the BT group and the UT group, there was no statistically significant difference. The FMA was used to assess the harm to bodily function and structure, making it the main indicator for evaluating the hemiplegic upper limbs. However, the FMA is not able to adequately evaluate the high-level muscular function of the distal upper extremities [58]. The WMFT, ARAT, and BBT were designed to evaluate the patient’s performance on a variety of activities, including those requiring fine motor control of the arms, hands, and fingers, as well as the overall quality of their performance, including the presence or absence of abnormalities in their stance and rate of movement. Increases in FMA-UE are associated with increases in the overall functional ability of the upper extremity, though this relationship is not straight. The FMA-UE has modest to high correlations with several other measures, including the WMFT, the ARAT, and the BBT [59,60,61]. Studies have shown that BT contributes very little to the functional precision of the hemiplegic hand and that the opposite brain hemisphere’s corticospinal cord is mainly responsible for directing minute motions of the distal upper limb [62]. These findings lend support to the idea that the brain hemisphere not engaged in action is the primary control of the associated movement, which is one potential explanation for our research findings. More studies are required to fully untangle the variables that are added to the links.

There appears to be a link between the length of time spent in therapy and the extent of healing in the extended period following a stroke. This was the finding of meta-analyses that compared the efficacy of different treatments based on the amount of time and energy each required. However, because patients and doctors place a higher emphasis on regaining mobility after a stroke than they do on regaining function in the upper extremities, UE training is frequently reduced to less than 10 min per therapy session. Therefore, any UE exercise that is active in addition to the current routine would be beneficial and in line with the most recent guidelines for the practice of strokes. Our study’s control group was engaged in exergaming activities at a frequency of at least twice weekly throughout the whole study. After only 21 min of training from their clinicians, they were able to exercise their upper limbs for a total of 45 min during each exergaming practice.

The study shows that using exergames is not only effective but also completely safe. Users of the exergame gadget reported feeling a mild degree of fatigue after prolonged use. No accidents were reported as a consequence of the falling that occurred during this seated exercise regimen. With the assistance of exergame devices, the amount of upper-limb (UE) therapy activities a stroke patient completes can be raised. The complexity of an operation can be increased by using exergames or any of the other many options accessible. For instance, one of the nearby recovery centers has outfitted a specific area with exergames, allowing patients to engage in productive play for prolonged amounts of time. The inclusion of fitness and play spaces in recovery settings has the potential to be a cost- and time-effective use of available resources.

### 6.1 Direction for future research and practice

The discussion showed that the BULT of stroke patients with hemiplegia was worth learning and promoting. The basis for patients to return to family and society was to improve the independence of ADLs in their daily life. For many ADLs, both hands were required to enable cooperation and coordination of actions to perform complementary tasks [63]. Short-term BULT can improve the local function of the affected upper limb to some extent, but it might not reach the threshold of muscle movement required to perform functional tasks [17,64]. The rehabilitation resources were relatively concentrated in the rehabilitation departments of comprehensive hospitals or specialized rehabilitation hospitals, but the length of a hospital stay is limited. After discharge, stroke patients failed to receive effective continuous rehabilitation training in their communities [18].

The purpose of this article is to propose some fresh avenues of research. Patients recovering motor function in their upper limbs following a stroke-related injury or amputation were the focus of the BULT program [16,20]. Workouts for the upper body were made easier under the UULT setting. For hemiplegic individuals, it is possible that in the future, BT and UT may become more tightly associated [21]. The high usability and robust practicality of BULT were not constrained by external variables like site or equipment, unlike previous systems. While BULT served a purpose in the short term, it was ultimately inefficient. Therefore, BULT may be conducted as soon as possible with assistance, taking into consideration the degree of hemiplegia in the affected upper limb, provided the patient is stable and willing. Only doing the process will not get the desired results [24,65]. Based on in-hospital rehabilitation training, patients should continue to perform BULT after returning to the community to improve their upper-limb function and ADLs. Future studies should explore the feasibility and effectiveness of continuous nursing care and compare the long-term effects. In addition, the dose of BULT was not directly proportional to the upper-limb function in post-stroke hemiplegia [66]. The intensity and time of exercise training must also be determined based on the patient’s tolerance and fatigue index. Moreover, the cooperation, interest, and willingness of patients in each rehabilitation training must be considered. Implementing the optimal effective dose in BULT was recommended to save rehabilitation resources and implement continuous nursing, promoting the maximum rehabilitation training benefits. Multi-center randomized controlled studies are encouraged to investigate the benefits of combining BULT and UULT for patients with stroke hemiplegia (RCTs) [67].

### 6.2 Strengths and limitations of this study

By determining the optimum scheduling and length of the intervention and the least effective daily training dosage, this study departs from the previous systematic studies that concentrated on the effectiveness of BT. This article can be used as a guide when thinking about how to reduce costs associated with rehabilitation, how to execute round-the-clock care, and how to advertise the greatest possible advantage of rehabilitation training. Patients originate from eight various nations and areas, all with varying medical standards, so study findings may not generalize to other cultural and regional groups. Significant variation in various outcome indicators may have been caused, at least in part, by the potential for bias presented by the small sample size and study population. Only a limited number of articles report on long-term follow-up data, and there is no systematic classification of stroke damage locations. In the meantime, the long-term impacts and implications of various tumor locations remain unknown.

## 7 Conclusion

The researcher says that the study’s goal is to determine how well BULT helps people who have experienced a stroke or other nerve disease recover movement in their upper limbs. Discourse and investigation of the past with the intent of clarifying the antecedents of the present. Data from all administered assessments, such as theARAT, BBT, WMFT, Fugl-Meyer Evaluation, and others, were considered. The researchers in this investigation read and analyzed about 24,000 works of writing. The data clearly show that the FMA’s safeguards fall short in important respects. Extreme Disruption Patients with hemiplegia who undergo therapy with BULT report feeling happier with their lives. Proponents of BULT exercise argue that even brief bouts of physical activity, such as 30 min, 5 days a week, for 6 weeks, can have positive effects. The meta-analysis included information from ten RCTs involving a total of 1,411 individuals. This meta-analysis found that when it came to decreasing lower limb muscle strain, overall spasm scale score, electromyography score, and passive ankle joint range of motion, biofeedback was significantly more effective than traditional physical therapy. A review of the research found that BULT helped individuals with stroke and hemiplegia use their affected limbs more effectively, but that it didn’t offer any advantages over solo training. It is most important to act quickly after a stroke, within the first 6 months. More research is required to demonstrate that BULT is helpful because acute trials were neglected. Thirty minutes of BULT exercise, twice a day, for 6 weeks was determined to be the minimally effective dosage. A relatively small number of people participated, and the evidence was only marginally better than usual. Patients suffering from stroke may benefit more from a therapy approach that combines BULT and UULT, but this debate should wait until more robust RCTs have been performed.

## 8 Future scope

Individuals participating in the exergaming recovery trial had to be fully functional 1 year after a stroke, without showing signs of the usual secondary disabilities. Possible causes for the patient’s difficulties include hemianopia, hemineglect, or structural abnormalities in the brain. As a result, we cannot generalize the study’s results to include all stroke victims worldwide. If researchers are intending on conducting bigger investigations in the future, they would do well to include people with a wide range of clinical traits. After it became clear that patients could not be kept safe throughout the process, a single-blind strategy was adopted. One of the drawbacks of this study is that it does not evaluate the patients’ views and degrees of happiness with the exergaming system. Future studies must account for the potential bias brought by this form of data when reporting their findings.
